# Polyphenol and flavonoid profiles and radical scavenging activity in leafy vegetable *Amaranthus gangeticus*

**DOI:** 10.1186/s12870-020-02700-0

**Published:** 2020-11-02

**Authors:** Umakanta Sarker, Shinya Oba

**Affiliations:** 1 Department of Genetics and Plant Breeding, Faculty of Agriculture, Bangabandhu Sheikh Mujibur Rahman Agricultural University, Gazipur-1706, Bangladesh; 2 Laboratory of Field Science, Faculty of Applied Biological Sciences, Gifu University, Yanagido 1-1, Gifu, Japan

**Keywords:** *A. gangeticus*, Phenolic profiles, Flavonoids, Antioxidant activity, HPLC-UV, LC-MS-ESI, DPPH and ABTS assays

## Abstract

**Background:**

Red amaranth (*Amaranthus gangeticus* L*.*) has great diversity in Bangladesh, India, and South East Asia with multipurpose uses. The bright red-violet colored *A. gangeticus* is a popular and low-cost leafy vegetable in the Asian continent including Bangladesh and India because of attractive leaf color, taste, adequate nutraceuticals, phenolic compounds, and sole source of betalains. The natural colors and phenolic compounds of this species have a significant role in promoting the health-benefit including the scavenging capacity of radicals, the colorant of food products, and play a vital role in the industry of foods. However, phenolic profiles and radical scavenging activity of this species have not been evaluated**.** Hence, for the first time, four selected advance lines of *A. gangeticus* were characterized for phenolic profiles, antioxidant constituents, and antioxidant potentiality.

**Results:**

*A. gangeticus* genotypes are abundant sources of phenolic profiles and antioxidant constituents with good radical quenching capacity that differed across the genotypes. Twenty-five phenolic acids and flavonoids, such as protocatechuic acid, salicylic acid, gentisic acid, gallic acid, β-resorcylic acid, vanillic acid, *p*-hydroxybenzoic acid, chlorogenic acid, ellagic acid, syringic acid, ferulic acid, kaempferol, *m*-coumaric acid, *trans*-cinnamic acid, quercetin, *p*-coumaric acid, apigenin, caffeic acid, rutin, sinapic acid, isoquercetin, naringenin, myricetin, catechin, and hyperoside were identified in *A. gangeticus* accessions. *A. gangeticus* accessions LS7 and LS9 demonstrated ample phenolic acids, flavonoids, antioxidant constituents, and antioxidant potentiality. It revealed from the correlation study that antioxidant components of *A. gangeticus* genotypes exhibited good radical scavenging activities. The genotypes LS7 and LS9 could be directly used as phenolic profiles, antioxidant constituents, and antioxidant activity enrich cultivars.

**Conclusions:**

The identified compounds of phenolic acids and flavonoids in *A. gangeticus* privilege the comprehensive study of pharmacology. The basic information on phenolic profiles and antioxidant constituents achieved in the present study will provide the scientist’s forum for the scientific assessment of these compounds in *A. gangeticus*.

## Background

The genus *Amaranthus* is a fast-growing C_4_ plant with versatile uses such as ornamental plants, vegetables, and grains. It has wider acclimatization and distributed worldwide including Africa, America, Asia, Australia, and Europe. Edible stems and leaves of different *Amaranthus* species are cheap vegetables containing good protein with lysine and methionine, dietary fiber [1–3], vitamins [4, 5], carotenoids, and minerals [6–8]. It also contains natural pigments [9–11]; natural phytochemicals, such as flavonoids, phenolic acids, and vitamins [12–15]. The above compounds have remarkable contributions to the industry of food as these compounds quench reactive oxygen species (ROS) in the human body and give protection against several diseases including neurodegenerative diseases, cancer, cardiovascular diseases, cataracts, emphysema, retinopathy, atherosclerosis, and arthritis [16–20]. These compounds also have a significant role in promoting the health-benefit and food colorants [21]. The species of this genus are tolerant of salinity [22–24] and drought stress [25–28].

*Amaranthus gangeticus* has high diversity in Bangladesh, India, and Asia [29] with multiple utilities. The selected genotypes are bright red-violet and maroon color due to the presence of abundant betalain. It is an inexpensive and famous leafy vegetable in the Asian continent including Bangladesh and India because of attractive leaf color, taste, and high nutritional value. In Bangladesh, *A. gangeticus* is grown throughout the year including a period of scarcity of leafy vegetables from the end of winter to the start of summer [1, 2]. *A. gangeticus* leaves inhibited the proliferation of colon (Caco-2) cancer cell lines, breast (MCF-7), and liver (HepG2) and exhibited anticancer potential [30].

Plants can be successfully engineered as biofactories for synthesizing biomolecules such as phenolic compounds, antioxidants, flavonoids, and vitamins having industrial and pharmaceutical interest; these efforts need the optimization of the above-mentioned biochemical knowledge to improve large-scale production, streamline the development of new products and make agribusiness increasingly competitive [31]. In the current decades, researches on food science are focused on polyphenols of plant origin, their antioxidant potentiality, accessibility in diets, and roles of protecting deadly diseases such as cardiovascular diseases, cancer, and neuro-degenerative [32]. Phenolic compounds are plant substances that possess in a common and aromatic ring bearing one or more hydroxyl groups. Flavonoids are the largest group of naturally occurring phenolic compounds, which occur in different plant parts both in a free state and as glycosides [33, 34]. Antioxidants of natural origin, such as phenolic components and vitamins are available in fruits and vegetables and protect against several diseases [35]. Phenolic components of plant origin can be classified into phenolic acids or simple phenols (hydroxycinnamic acids and hydroxybenzoic acids), tannins, flavonoids, and lignins that are involved in antioxidant potentiality, bitterness, color, flavor, acerbic taste, and odorness [36]. Antioxidant compounds reduce oxidative damage to the human body through inhibition of the oxidizing chain reactions caused by free radical [37]. Flavonoids and phenolic compounds are natural antioxidants, which can act as free radical scavengers [34]. Quercetin quenches free radicals to prevent the oxidation of low-density lipoprotein [38]. Ellagic acid has health-promoting effects due to its anticarcinogenic and antimutagenic responses [39].

Total phenolic content and antioxidant activity of *A. gangeticus* have already been reported [40]. However, there is little information available in the literature regarding the phenolic composition of this plant. Currently, the research group is evaluating the chances of utilizing *A. gangeticus* phenolic profiles, antioxidant constituents, as it has abundant natural potent antioxidants of interest in the food industry [18, 20]. Based on yields and antioxidant activity, *A. gangeticus* genotypes were previously screened and the best four high yielding and antioxidant potential genotypes LS3, LS5, LS7, and LS9 were selected as advanced lines. It is the first attempt to study the phenolic profiles, antioxidant constituents, and antioxidant capacity in *A. gangeticus*. Hence, in this study, phenolic profiles, antioxidant constituents, and antioxidant potentiality of selected four advanced lines of *A. gangeticus* leafy vegetables were characterized in detail using high-performance liquid chromatography (HPLC) and liquid chromatography-mass spectrometry (LC-MS). The results of the present study improve the understanding of vitamins, phenolic, and flavonoids compounds and the antioxidant potentiality of *A. gangeticus* leafy vegetables for the food industry, nutritionists, pharmacists, and consumers.

## Methods

### Experimental materials

The seeds of four advance genotypes were collected from the Department of Genetics and Plant Breeding.

### Design and layout

A completely randomized block design (RCBD) was used to execute the experiment at Bangabandhu Sheikh Mujibur Rahman Agricultural University with three replicates. The seeds of each genotype were sown in the ambient field of 1 m^2^ experimental plot following 20 cm and 5 cm distance between rows and plants, respectively.

### Climatic and edaphic conditions and intercultural practices

The site falls under the subtropical zone and has mean temperatures of 29 °C (summer) and 18 °C (winter). There was no precipitation during the cropping season. The soil characteristics are silty clay, a little acidic (pH 6.4) with low content of organic matter (0.87%). The compost (10 ton/ha) was applied at the time of preparation of lands. Gypsum, MP, TSP, and Urea were applied @ 30, 150, 100, and 200 kg/ha, respectively [41]. Proper intercultural operations were continued. For maintaining the exact spacing of plants in a row, proper thinning was executed. Weeds of experimental plots were regularly removed through proper weeding and hoeing. Regular irrigation was provided in the experimental plots for maintaining the proper growth of leafy vegetable amaranth. The leaf samples were collected from 10 randomly selected plants of each experimental plot at 30 days old plant (vegetative stage).

### Solvents and reagents

Methanol, acetic acid (HPLC grade), acetonitrile (HPLC grade), acetone, standard phenolic compounds, 2, 2-dipyridyl, dithiothreitol (DTT), DPPH (2, 2-diphenyl1-picrylhydrazyl), standard Trolox, ABTS, gallic acid, aluminum chloride hexahydrate, Folin-Ciocalteu reagent, potassium acetate, rutin, sodium carbonate, and potassium persulfate. All solvents and reagents were bought from Merck (Germany) and Kanto Chemical Co. Inc. (Tokyo, Japan).

### Samples extraction for HPLC and LC-MS analysis

The leaf samples were extracted by adding 10 mL methanol (80%) containing acetic acid (1%) in 1 g of leaves. The mixture was thoroughly homogenized. Then the mixture was kept in a test tube (50 mL) and capped tightly. The test tube was shaken in a shaker (Scientific Industries Inc., USA) for 15 h at 400 rpm. Finally, the extract was centrifuged for 15 min at 10,000×g and filtered through a 0.45 μm filter. The phenolic compounds were analyzed from the final filtrate. All extractions were performed in triplicate independent samples.

### Determination of phenolics through HPLC

The HPLC method previously described by Sarker & Oba [11, 28] was used to determine phenolic profiles in *A. gangeticus* leaf samples. Shimadzu SCL10Avp (Kyoto, Japan) HPLC was equipped with a binary pump (LC-10Avp), a degasser (DGU-14A), and a Shimadzu detector (SPD-10Avp UV–Vis). Flavonoids and phenolic acids were separated using a column [CTO-10 AC (STR ODS-II, 150 × 4.6 mm I.D., (Kyoto, Japan)] [11]. The binary mobile phase was pumped with solvent A (6% v/v acetic acid) in water and solvent B (acetonitrile) at the flow rate of 1 mL/min for 70 min. HPLC system was run using a gradient program with 0–15% acetonitrile for 45 min, 15–30% for 15 min, 30–50% for 5 min, and 50–100% for 5 min. Thirty-five °C temperature in the column was maintained with a 10 μL volume of injection [11]. The detector was set at 360, 370, 280, and 254 nm, respectively for continuous monitoring of flavonoids, cinnamic acids, and benzoic acids. For identification of the compounds, retention time and UV–vis spectra were compared with their respective standards. The confirmation of flavonoids and phenolic acids were performed through the mass spectrometry assay method. HPLC detected total compounds were represented as a total phenolic index (TPI). The previously described method of Sarker & Oba [11, 28] was used to calculate TPI from the HPLC data. All samples were prepared and analyzed in duplicate. Phenolic compounds were estimated as μg g^− 1^ FW. A mass spectrometer (AccuTOF, Japan) equipped with HPLC (Agilent 1100 Series) and a detector (UV–Vis) attached on-line with an ESI to analyze the spectrometric masses in negative ion mode maintaining the column elutes range of m/z 0–1000 and needle voltage at − 2000 V. Extract constituents were identified by LC-MS-ESI analysis.

### Quantification of phenolic compounds

The respective standards of calibration curves were used to quantify each phenolic compound. We dissolved 25 phenolic compounds in 80% methanol as stock solutions to the final concentration of 100 mg/mL. Respective standard curves (10, 20, 40, 60, 80, and 100 mg/mL) were used to quantify the individual phenolic compounds with external standards. UV spectral characteristics, retention times, and co-chromatography of samples spiked with commercially available standards were applied for identification and match the phenolics. The phenolic compounds were quantified estimating the area of peak of corresponding standards.

### Estimation of pro-vitamin A

The pro-vitamin A was estimated following our previously described method [7, 14]. The fresh leaf samples (0.5 g each) were extracted with adding acetone (10 mL, 80%) followed by centrifugation for 3–4 min at 10,000 × g. A spectrophotometer was set at 510 nm and 480 nm, respectively to read the absorbance. The results were calculated as mg pro-vitamin A per 100 g FW.

$$ \mathrm{Pro}-\mathrm{vitaminA}=\left\{7.6\ \left({\mathrm{A}}_{480}\right)-1.49\ \left({\mathrm{A}}_{510}\right)\right\}\times \mathrm{Final}\ \mathrm{volume}/\left\{1000\times \mathrm{weight}\ \mathrm{of}\ \mathrm{leaf}\ \left(\mathrm{fresh}\right)\right\} $$

### Estimation of vitamin C

Vitamin C was estimated following our previously described method [14, 42] by pre-incubation of samples using Dithiothreitol. Ascorbate reduced ferric ion to ferrous ion. 2, 2-dipyridyl bound with reduced ferrous ion to form complexes. A spectrophotometer was set at 525 nm to read the absorbance of Fe^2+^ complexes. Vitamin C was calculated in mg 100 g^− 1^ FW.

### Extraction of samples for TP, TF, and TAC

*A. gangeticus* leaves were collected at 30 days old plant. The collected leaves were air-dried in a shady place. 40 mL methanol (90%) was utilized to extract samples from 1 g of fresh leaves (for TF) and dried leaf powder (for TF and TAC) of each accession in a capped test tube. The test tubes were shaken in a Thomastant T-N22S (Japan) water bath shaker for 1 h. Finally, the extract was centrifuged for 15 min at 10,000×g and filtered through a 0.45 μm filter. The TAC, TF, and TP were estimated from the filtered extract.

### Estimation of total polyphenols

The estimation of total polyphenols was carried out according to Sarker & Oba [43, 44] and Jimenez-Aguilar & Grusak [45] using Folin-Ciocalteau reagent. A microplate reader was set to detect the optical density at 740 nm. The results were estimated as μg GAE g^− 1^ FW.

### Estimation of total flavonoids

Estimation of flavonoids was carried out according to the method described by Jimenez-Aguilar & Grusak [45] using AlCl3. A microplate reader was set to detect the optical density at 500 nm. The results were estimated as μg RE g^− 1^ DW.

### Radical quenching capacity assay

The antioxidant activity was estimated following the DPPH radical quenching assay [46, 47] and the ABTS method [48]. The absorbance was read at 517 (DPPH) and 734 (ABTS) nm using a Hitachi spectrophotometer (Japan). The antioxidant capacity (ABTS and DPPH) was measured according to the following equation:

$$ \mathrm{AC}\ \left(\%\right)=\left({\mathrm{A}}_{\mathrm{b}}-{\mathrm{A}}_{\mathrm{s}}/{\mathrm{A}}_{\mathrm{b}}\right)\times 100 $$

Where, AC = antioxidant capacity, A_b_ = absorbance of the blank sample [10 and 150 μL methanol for DPPH and ABTS, respectively as a substitute of leaf extract], and A_s_ = absorbance of the test compound. The results were calculated as μg TEAC g^− 1^ DW.

### Statistical analysis

The analysis of ANOVA was performed from mean data using software namely Statistix 8 [49, 50]. The Mean separation was carried out following DMRT (*P* < 0.01). The results indicated as mean ± SD.

## Results

### Flavonoids and phenolic acids

The data on main fragment ions in MS^2^, identified compounds, λmax, the molecular ion, and retention time are presented in Table 1. The flavonoids and phenolic acid data from four genotypes (LS3, LS5, LS7, and LS9) separated by liquid chromatography were compared with standard masses of phenolics and their respective peaks. Twenty-five flavonoid and phenolic acids such as protocatechuic acid, vanillic acid, gallic acid, salicylic acid, gentisic acid, *p*-hydroxybenzoic acid, β-resorcylic acid, syringic acid, ellagic acid, *m*-coumaric acid, *trans*-cinnamic acid, caffeic acid, chlorogenic acid, ferulic acid, sinapic acid, *p*-coumaric acid, rutin, naringenin, kaempferol, myricetin, catechin, isoquercetin, apigenin, hyperoside, and quercetin were detected in *A. gangeticus*. Across the compounds, seven compounds were identified as cinnamic acids, nine compounds were identified as benzoic acids, and nine compounds were identified as flavonoids compounds. Concerning three main classes of phenolics, the most prominent compounds were identified in four advance lines of *A. gangeticus* genotypes in the order: benzoic acids > cinnamic acids > flavonoids.

**Table 1:** Retention time (Rt), wavelengths of maximum absorption in the visible region (λ_max_), mass spectral data, and tentative identification of phenolic compounds in four selected *A. gangeticus* leafy vegetables

Peak no	Rt (min)	λ_max_ (nm)	Molecular ion [M - H]^−^ (m/z)	MS^2^ (m/z)	Identity of tentative compounds
1	9.12	254	169.1354	169.1465	3,4,5 Trihydroxybenzoic acid
2	30.61	254	167.1423	167.1634	4-Hydroxy-3-methoxy benzoic acid
3	34.82	254	197.1125	197.1125	3,5-Dimethoxy-4-hydroxybenzoic acid
4	31.52	254	137.0325	137.0425	4-Hydroxybenzoic acid
5	48.23	254	137.2216	137.3241	2-Hydroxybenzoic acid
6	52.51	254	301.0384	301.0586	2,3,7,8-Tetrahydroxy-chromeno [5,4,3-cde] chromene-5,10-dione
7	2.24	280	154.1342	154.1452	3,4-Dihydroxybenzoic acid
8	4.13	280	154.1354	154.1387	2,4-Dihydroxybenzoic acid
9	3.71	280	154.1357	154.1456	2,5- Dihydroxybenzoic acid
10	32.12	280	179.0792	179.0748	3,4-Dihydroxy-trans-cinnamate
11	31.14	280	353.1352	353.1453	3-(3,4-Dihydroxy cinnamoyl) quinic acid
12	42.16	280	163.0586	163.1105	4-Hydroxy cinnamic acid
13	47.95	280	193.1843	193.1748	3-Methoxy-4-hydroxy cinnamic acid
14	49.61	280	163.2621	163.2784	3-Hydroxy cinnamic acid
15	49.14	280	223.1632	223.1752	4-Hydroxy-3,5-dimethoxy cinnamic acid
16	67.32	280	147.1214	147.1143	3-Phenyl acrylic acid
17	23.93	280	290.2368	290.2246	(2R-3S)-2-(3,4-dihydroxyphenyl)-3,4-dihydro-2-chromene-3,5,7-triol
18	26.73	280	271.0746	271.1527	Naringenin
19	54.35	360	463.2795	463.3345	Quercetin-3-*O*-glucoside
20	53.32	360	463.4428	463.5243	Quercetin-3-*O*-galactoside
21	53.34	360	609.3788	609.3687	Quercetin-3-*O*-rutinoside
22	7.56	370	301.0425	301.0348	2-(3,4-dihydroxy phenyl)-3,5,7-trihydroxychromene-4-one
23	4.61	370	626.1954	626.2675	Myricetin-3-*O*-rutinoside
24	15.45	370	270.3426	270.3548	4′,5,7-Trihydroxyflavone, 5,7-Dihydroxy-2-(4-hydroxyphenyl)-4-benzopyrone
25	17.82	370	593.5214	593.3264	kaempferol-3-*O*-rutinoside

### Benzoic acids

**Fig. 1:**
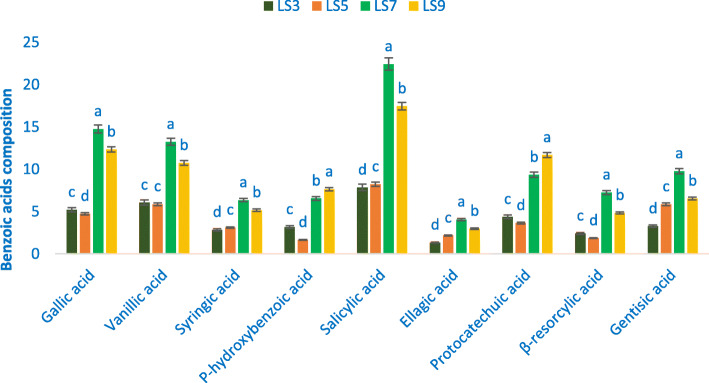
Benzoic acids profile (μg g^− 1^ FW) in four selected *A. gangeticus* leafy vegetables, in the bar different letters are significantly differed by DMRT, (*n* = 3), (*P* < 0.01)

The most available phenolic acids were salicylic acids. The rest of the benzoic acids were identified in the following order: ellagic acid < syringic acid < β-resorcylic acid *p*-hydroxybenzoic acid < gentisic acid < protocatechuic acid < vanillic acid < gallic acid (Fig. 1).

The range of vanillic acid, gentisic acid, salicylic acid, gallic acid, β-resorcylic acid, syringic acid, and ellagic acid were 5.88 to 13.24, 3.27 to 9.78, 7.87 to 22.43, 4.74 to 14.76, 1.86 to 7.26, 2.85 to 6.36, and 1.35 to 4.08 μg g^− 1^ FW, individually (Fig. 1). The maximum vanillic acid (13.24 μg g^− 1^ FW), gentisic acid (9.78 μg g^− 1^ FW), salicylic acid (22.43 μg g^− 1^ FW), gallic acid (14.76 μg g^− 1^ FW), β-resorcylic acid (7.26 μg g^− 1^ FW), syringic acid (6.36 μg g^− 1^ FW), and ellagic acid (4.08 μg g^− 1^ FW) were recorded in LS7. In contrast, the genotype LS5 showed the lowest vanillic acid (5.88 μg g^− 1^ FW), gallic acid (4.74 μg g^− 1^ FW), and the genotype LS3 had the lowest salicylic acid (7.87 μg g^− 1^ FW), gentisic acid (3.27 μg g^− 1^ FW), β-resorcylic acid (1.86 μg g^− 1^ FW), syringic acid (2.85 μg g^− 1^ FW), and ellagic acid (1.35 μg g^− 1^ FW). Protocatechuic acid and *p*-hydroxybenzoic acid ranged from 3.65 to 11.68 and 1.65 to 7.64 μg g^− 1^ FW, individually (Fig. 1). LS9 demonstrated the maximum protocatechuic acid (11.68 μg g^− 1^ FW) and *p*-hydroxybenzoic acid (7.64 μg g^− 1^ FW), whereas, LS5 demonstrated the minimum protocatechuic acid (3.65 μg g^− 1^ FW) and *p*-hydroxybenzoic acid (1.65 μg g^− 1^ FW).

### Cinnamic acids

**Fig. 2:**
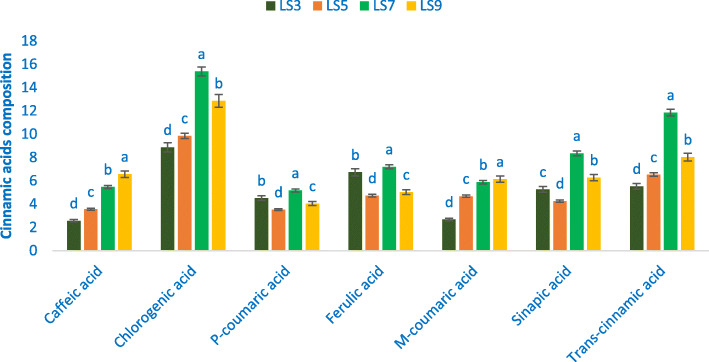
Cinnamic acids profile (μg g^− 1^ FW) in four selected *A. gangeticus* leafy vegetables, in the bar different letters are significantly differed by DMRT, (*n* = 3), (*P* < 0.01)

Chlorogenic acid was identified as the most prominent compound within cinnamic acids followed by *trans*-cinnamic acid, sinapic acid, and ferulic acid (Fig. 2). *A. gangeticus* genotypes demonstrated ample cinnamic acids. The range of chlorogenic acid, *trans*-cinnamic acid, sinapic acid, *p*-coumaric acid, and ferulic acid was 8.86 to 15.38, 5.52 to 11.85, 4.26 to 8.35, 4.72 to 7.20, and 3.51 to 5.16 μg g^− 1^ FW, individually (Fig. 2). The maximum *trans*-cinnamic acid, chlorogenic acid, sinapic acid, ferulic acid, and *p*-coumaric acid (15.38, 11.85, 8.35, 7.20, and 5.16 μg g^− 1^ FW, individually) were noted in LS7. Whereas, LS3 exhibited the minimum chlorogenic acid and *trans*-cinnamic acid (8.86 and 5.52 μg g^− 1^ FW) and LS5 demonstrated the minimum sinapic acid, ferulic acid, and *p*-coumaric acid (4.26, 4.72, and 3.51 μg g^− 1^ FW). The range of *m*-coumaric acid and caffeic acid was 2.67 to 6.13 and 2.56 to 6.65 μg g^− 1^ FW (Fig. 2). The maximum *m*-coumaric acid and caffeic acid (6.13 and 6.65 μg g^− 1^ FW) were recorded in LS9. Conversely, LS3 had the lowest *m*-coumaric acid and caffeic acid (2.67 and 2.56 μg g^− 1^ FW).

### Flavonoids

**Fig. 3:**
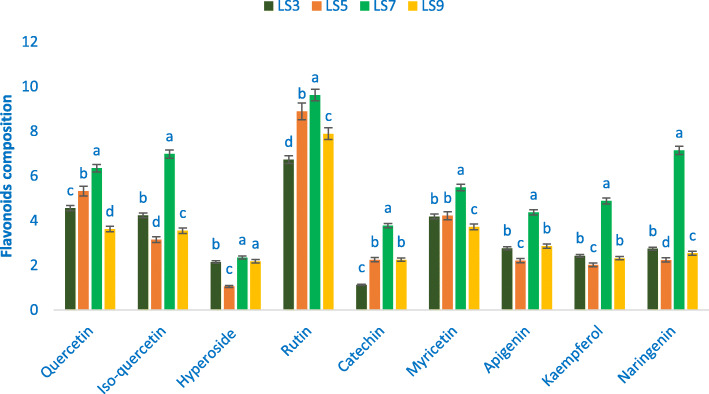
Flavonoids profile (μg g^− 1^ FW) in four selected *A. gangeticus* leafy vegetables, in the bar different letters are significantly differed by DMRT, (*n* = 3), (*P* < 0.01)

In this study, *A. gangeticus* genotypes demonstrated ample flavonoids such as rutin, naringenin, isoquercetin, quercetin, myricetin, kaempferol, apigenin, hyperoside, and catechin. Rutin, naringenin, isoquercetin, quercetin, myricetin, kaempferol, apigenin, hyperoside, and catechin varied from 6.73 to 9.62, 2.24 to 7.14, 3.15 to 6.98, 3.62 to 6.35, 3.72 to 5.48, 2.02 to 4.88, 2.21 to 4.37, 1.05 to 2.35, and 1.12 to 3.78 μg g^− 1^ FW, individually (Fig. 3). The accession LS7 demonstrated the maximum rutin, naringenin, isoquercetin, quercetin, myricetin, kaempferol, apigenin, hyperoside, and catechin (9.62, 7.14, 6.98, 6.35, 5.48, 4.37, 2.35, and 3.78 μg g^− 1^ FW, respectively). Hyperoside content of LS7 had statistical similarity with LS9. The accessions LS5 and LS3 demonstrated high myricetin (4.22 and 4.18 μg g^− 1^ FW), the accessions LS5 and LS9 demonstrated high rutin (8.89 and 7.89 μg g^− 1^ FW), the accessions LS5 demonstrated high quercetin (5.32 μg g^− 1^ FW), and the accession LS3 demonstrated high isoquercetin and hyperoside (4.23 and 2.15 μg g^− 1^ FW). In contrast, the accession LS9 demonstrated the minimum quercetin and myricetin (3.62 and 3.72 μg g^− 1^ FW), the accession LS3 exhibited the minimum rutin and catechin (6.73 and 1.12 μg g^− 1^ FW), and the accession LS5 exhibited the minimum isoquercetin, naringenin, apigenin, kaempferol, and hyperoside (3.15, 2.24, 2.21, 2.02, and 1.05 μg g^− 1^ FW, respectively) (Fig. 3).

### Phenolic fractions

**Fig. 4:**
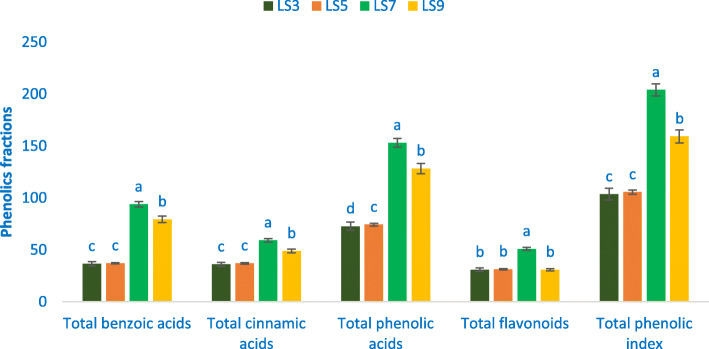
Phenolic fractions composition (μg g^− 1^ FW) in four selected *A. gangeticus* leafy vegetables, in the bar different letters are significantly differed by DMRT, (*n* = 3), (*P* < 0.01)

The range of total phenolic index (TPI), total cinnamic acids (TCA), total benzoic acids (TBA), total flavonoids (TF), and total phenolic acids (TPA) were 103.58 to 204.03, 36.10 to 59.27, 36.59 to 93.81, 30.89 to 50.95, and 72.69 to 153.08 μg g^− 1^ FW, individually (Fig. 4). The accession LS7 showed the maximum TPI (204.03 μg g^− 1^ FW), TCA (59.27 μg g^− 1^ FW), TBA (93.81 μg g^− 1^ FW), TPA (153.08 μg g^− 1^ FW), and TF (50.95 μg g^− 1^ FW). The TF of the accession LS3 and LS5 had statistical similarity to the accession of LS9. Conversely, the accessions LS3 and LS5 demonstrated the minimum TF (30.89, 31.35 μg g^− 1^ FW), TCA (36.10, 37.10 μg g^− 1^ FW), TBA (36.59, 37.18 μg g^− 1^ FW), and TPI (103.58, 105.63 μg g^− 1^ FW). The accession LS3 demonstrated the minimum TPA (72.69 μg g^− 1^ FW) (Fig. 4).

### Antioxidant constituents and radical quenching capacity

**Fig. 5:**
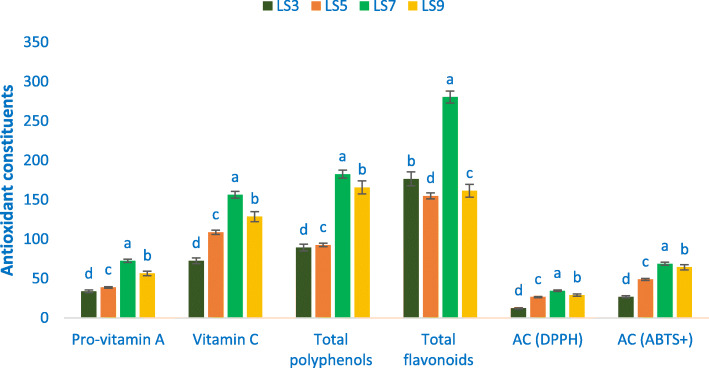
Antioxidant constituents and quenching ability of free radicals in four selected *A. gangeticus*, Pro-vitamin A (mg 100 g^− 1^ FW), TP (μg GE g^− 1^ FW), Vitamin C (mg 100 g^− 1^ FW), TF (μg RE g^− 1^ DW), AC (DPPH) = antioxidant capacity (DPPH) (μg TEAC g^− 1^ DW), AC (ABTS) = antioxidant capacity (ABTS) (μg TEAC g^− 1^ DW), in the bar different letters are significantly differed by DMRT, (*n* = 3), (*P* < 0.01)

TP, pro-vitamin A, vitamin C, AC, and TF differed significantly regarding the advance lines of *A. gangeticus* accession (Fig. 5). The range of pro-vitamin A content was 33.62 mg 100 g^− 1^ in LS3 to 72.34 mg 100 g^− 1^ in LS7. The accession LS7 demonstrated the maximum pro-vitamin A and LS9 exhibited high pro-vitamin A content. The range of vitamin C content was 72.45 mg 100 g^− 1^ in LS3 to 156.34 mg 100 g^− 1^ in LS7. The range of TP was 89.34 μg g^− 1^ (LS3) to 182.55 μg g^− 1^ (LS7). The accession LS7 exhibited the maximum total polyphenols followed by LS9. TF exhibited prominent variation regarding genotypes with a range of 154.89 μg g^− 1^ in LS5 to 280.44 μg g^− 1^ in LS7. The range of antioxidant capacity (DPPH) was 12.27 μg g^− 1^ (LS3) to 34.38 μg g^− 1^ (LS7). The maximum antioxidant capacity (DPPH) was recorded in LS7 followed by LS9 and LS5. In contrast, LS3 demonstrated the minimum antioxidant capacity (DPPH). The range of antioxidant capacity (ABTS) was 26.69 μg g^− 1^ to 68.89 μg g^− 1^. The *A. gangeticus* advance line LS7 demonstrated the maximum antioxidant capacity (ABTS) followed by LS9. In contrast, antioxidant capacity (ABTS) was the minimum in LS3.

### Correlation coefficient study

The correlation of antioxidant constituents and the antioxidant capacity of *A. gangeticus* are shown in Table 2. Pro-vitamin A and vitamin C had significant positive interrelationships with vitamins, TP, TF, AC (DPPH and ABTS). A significant correlation among TP, TF, AC (DPPH and ABTS) were observed. Similarly, AC (ABTS) was significantly interrelated with AC (DPPH).

**Table 2:** The correlation coefficient for antioxidant constituents and radical quenching capacity in four selected *A. gangeticus* leafy vegetables

	Pro-vitamin A (mg 100 g^− 1^ FW)	Vitamin C (mg 100 g^− 1^ FW)	Total polyphenols (GAE μg g^− 1^ FW)	Total flavonoids (RE μg g^− 1^ DW)	AC (DPPH) (TEAC μg g^− 1^ DW)	AC (ABTS^+^) (TEAC μg g^− 1^ DW)
Pro-vitamin A		0.65^b^	0.74^b^	0.96^b^	0.53^a^	0.58^a^
Vitamin C			0.78^b^	0.85^b^	0.77^b^	0.78^b^
Total polyphenols				0.61^b^	0.88^b^	0.87^b^
Total flavonoids					0.58^a^	0.56^a^
AC (DPPH)						0.98^b^

*AC (DPPH)* Antioxidant capacity (DPPH), *AC (ABTS*^*+*^*)* Antioxidant capacity (ABTS^+^), ^a^significant at 5% level, ^b^significant at 1% level, (*n* = 3)

## Discussion

Vitamins including pro-vitamin A and Vitamin C, flavonoids, and polyphenol compounds from natural origins, such as vegetables and fruits serve as antioxidants and protect against several diseases [35]. Recently, food researchers and consumers are interested in vitamins, polyphenols, and flavonoids of plant origin, their antioxidant potentiality, accessibility in diets, and roles of protecting deadly diseases such as cardiovascular diseases, cancer, and neuro-degenerative [32]. Phenolic compounds are plant substances that possess in a common and aromatic ring bearing one or more hydroxyl groups. Flavonoids are the largest group of naturally occurring phenolic compounds, which occur in different plant parts both in a free state and as glycosides [33, 34].Antioxidants compounds reduce oxidative damage to the human body through inhibition of the oxidizing chain reactions caused by free radical [37]. The shikimic acid pathway transformed phenylalanine and tyrosine into phenolic acids and flavonoids in plants [51]. Flavonoids have important biological functions in the human body. Quercetin quenches free radicals to prevent the oxidation of low-density lipoprotein [38]. Ellagic acid has health-promoting effects due to its anticarcinogenic and antimutagenic responses [39].

The analysis of variance revealed a wide range of variability of the studied traits regarding selected advance genotypes of *A. gangeticus*. A wide range of variability of the studied traits was also reported in *A. tricolor* and *A. lividus* [11], rice [52–65], maize [66–68], and coconut [69]. By the liquid chromatography assay, twenty-five flavonoid and phenolic compounds such as protocatechuic acid, vanillic acid, gallic acid, salicylic acid, gentisic acid, *p*-hydroxybenzoic acid, β-resorcylic acid, syringic acid, ellagic acid, *m*-coumaric acid, *trans*-cinnamic acid, caffeic acid, chlorogenic acid, ferulic acid, sinapic acid, *p*-coumaric acid, rutin, naringenin, kaempferol, isoquercetin, myricetin, apigenin, catechin, quercetin, and hyperoside were identified in *A. gangeticus*. Across the compounds, seven compounds were identified as cinnamic acids, nine compounds were identified as benzoic acids, and nine compounds were identified as flavonoids compounds. In the previous study, 24 flavonoids and phenolic acids were identified in the leaves of red and green color amaranth *(A. tricolor* and *A. lividus*) [11]. Khanam et al. [48] and Khanam & Oba [70] identified 16 phenolic and flavonoid compounds such as syringic acid, vanillic acid, *m*-coumaric acid, gallic acid, salicylic acid, *p*-hydroxybenzoic acid, ellagic acid, caffeic acid, *trans*-cinnamic acid, chlorogenic acid, rutin, ferulic acid, *p*-coumaric acid, sinapic acid, hyperoside, and isoquercetin in green and red amaranths. In the stalks, leaf, sprouts, flowers, and the seed of *A. caudatus*, *A. cruentus*, and *A. hypochondriacus*, eleven phenolics including gallic acid, chlorogenic acid, ferulic acid, β-resorcylic acid, gentisic acid, salicylic acid, protocatechuic acid, ellagic acid, rutin, quercetin, and kaempferol were detected [71]. Eight phenolics like *p*-hydroxybenzoic acid, gallic acid, vanillic acid, *p*-coumaric acid, ferulic acid, cinnamic acids, caffeic acids, syringic acids, vitexin, rutin, and isovitexin were reported in *A. cruentus* seeds and sprouts [72]. Across the three main classes of phenolics, the most prominent compounds were identified in four advance lines of *A. gangeticus* genotypes in the following order: benzoic acids > cinnamic acids > flavonoids.

The most available phenolic acids were salicylic acids. The rest of the benzoic acids were identified in the following order: ellagic acid < syringic acid < β-resorcylic acid *p*-hydroxybenzoic acid < gentisic acid < protocatechuic acid < vanillic acid < gallic acid. We obtained much greater benzoic acid content in the *A. gangeticus* genotype LS7 and LS9 compared to the results of the benzoic acid content of green amaranth of our previous study [11] and the results of the benzoic acid content of *A. tricolor* [48]. The maximum salicylic acid, vanillic acid, gallic acid, gentisic acid, β-resorcylic acid, syringic acid, and ellagic acid were obtained from the genotype LS7 followed by the genotype LS9. The genotype LS9 exhibited the maximum protocatechuic acid and *p*-hydroxybenzoic acid followed by LS7. Hence, the selected advance genotypes LS7 and LS9 could be considered as high benzoic acid profiles enrich genotypes. These two genotypes could be directly used as benzoic acid profiles enrich cultivars.

Chlorogenic acid was identified as the most pronounced compound within cinnamic acids followed by *trans*-cinnamic acid, sinapic acid, and ferulic acid. *A. gangeticus* genotypes demonstrated ample cinnamic acids. Seven cinnamic acids obtained from the genotype LS7 and LS9 were much greater in comparison with the results of cinnamic acids in *A. tricolor* [48]. The maximum *trans*-cinnamic acid, chlorogenic acid, sinapic acid, ferulic acid, and *p*-coumaric acid were observed in LS7. The maximum *m*-coumaric acid and caffeic acid were reported in LS9. Seven cinnamic acids found in the current investigation were much greater in comparison to the seven cinnamic acids of green amaranth of our previous study [11]. The selected advance genotypes LS7 and LS9 could be considered as high cinnamic acid profiles enrich genotypes. These two genotypes could be directly used as cinnamic acids acid profiles enrich cultivars.

In this study, *A. gangeticus* genotypes demonstrated ample flavonoids such as myricetin, rutin, naringenin, isoquercetin, quercetin, kaempferol, apigenin, catechin, and hyperoside which were much greater in comparison to nine flavonoid compounds of green amaranth of our previous study [11]. LS7 demonstrated the maximum myricetin, rutin, naringenin, isoquercetin, quercetin, kaempferol, apigenin, catechin, and hyperoside. Quercetin and hyperoside of *A. gangeticus* accessions were greater in comparison to the content of quercetin and hyperoside reported in *A. tricolor* [48]. Hence, the selected advance accession LS7 could be considered as high flavonoids profiles enrich accession. This accession could be directly used as flavonoids profiles enrich cultivar.

LS7 demonstrated the maximum TCA, TBA, TPI, TPA, and TF. The TPI, TF, and TPA of *A. gangeticus* accessions were much pronounced in comparison to the content of *A. tricolor* [48]. Cinnamic acid was synthesized in the tissues of plants from the most widely available phenylalanine [73]. In the tissue of plants, although glycoside derivatives are the types of flavonoids which are most available and also occur as aglycone. Across total phenolics, 60% are represented as flavonoids [74]. Naturally, the most available flavonoids in the plants are flavonols and quercetin glycosides [74]. Different genotypes of the *Cichorium spinosum* exhibited significant differences in flavonoids and phenolic acid profiles [75].

In this study, considerable phenolics, such as gallic acid, protocatechuic acid, salicylic acid, vanillic acid, *trans*-cinnamic acid, *p*-hydroxybenzoic acid, gentisic acid, β-resorcylic acid, ellagic acid, syringic acid, caffeic acid, chlorogenic acid, *m*-coumaric acid, ferulic acid, sinapic acid, *p*-coumaric acid, rutin, isoquercetin, naringenin, quercetin, kaempferol, myricetin, catechin, apigenin, and hyperoside were identified in *A. gangeticus* genotypes. The reported results of Khanam & Oba [70] were corroborative to the results of our present study. They obtained greater vanillic acid, salicylic acid, gallic acid, ellagic acid, syringic acid, *trans*-cinnamic acid, *m*-coumaric acid, *p*-hydroxybenzoic acid, chlorogenic acid, caffeic acid, isoquercetin, ferulic acid, rutin, and *p*-coumaric acid in *A. tricolor* genotypes in comparison with green amaranth*.* In this study, obtained vanillic acid, salicylic acid, *p*- hydroxybenzoic acid, gallic acid, *m*-coumaric acid, syringic acid, *trans*-cinnamic acid, ellagic acid, chlorogenic acid, caffeic acid, *p*-coumaric acid, hyperoside, ferulic acid, and rutin were greater in comparison with the phenolic acids of *A. tricolor* [48]*. A. gangeticus* accession LS7 contained high flavonoids including rutin, naringenin, isoquercetin, quercetin, myricetin, kaempferol, apigenin, catechin, and hyperoside. The accessions LS7 and LS9 exhibited high phenolic profile including gallic acid, protocatechuic acid, vanillic acid, salicylic acid, *trans*-cinnamic acid, *p*-hydroxybenzoic acid, gentisic acid, ellagic acid, β-resorcylic acid, syringic acid, caffeic acid, *m*-coumaric acid, chlorogenic acid, ferulic acid, sinapic acid, *p*-coumaric acid. The accessions LS7 and LS9 might be utilized as high-yielding cultivars containing ample phenolics. It revealed from this study that the genotypes LS7 and LS9 containing abundant phenolic profiles demand deep and elaborate pharmacological study to find out the new insight of this crop.

TP, pro-vitamin A, vitamin C, AC, and TF differed significantly among the advance lines of *A. gangeticus*. The accession LS7 showed the maximum pro-vitamin A, vitamin C, TP, TF, AC (DPPH assay), and AC (ABTS assay) followed by LS9. Khanam & Oba [70] reported higher TP, TF, and AC content in *A. tricolor* genotypes in comparison with green amaranth which was corroborative to our present study. The *A. gangeticus* advance lines LS7 and LS9 contained high pro-vitamin A, vitamin C, TP, TF, and AC compared to the accession LS3 and LS5. Hence, these antioxidant constituents of *A. gangeticus* advance lines could be an important parameter for consumers, playing a vital contribution for quenching of ROS, protecting aging, and several degenerative human diseases [18, 20]. Our result revealed that the *A. gangeticus* accessions had an abundant source of antioxidant constituents such as pro-vitamin A, vitamin C, TF, TF, and AC across leafy vegetables that has important free radical-scavenging activity [21].

In this study, considerable antioxidant constituents such as pro-vitamin A, vitamin C, TP, TF, and AC were found in the *A. gangeticus* accessions. The findings of the present study were corroborative to the findings of TP, TP, and AC of *A. tricolor* [70]. AC (ABTS), TF, and AC (DPPH) obtained in this study corroborated with the findings of *A. tricolor* [48] whereas, TP obtained in this study were much greater in comparison to TP of *A. tricolor* [48]*.* Vitamin C obtained from *A. gangeticus* accessions was much greater in comparison to vitamin C of different *Amaranthus* species [45]. The present results showed that the red color *A. gangeticus* genotypes exhibited 2 to 3-fold higher β-carotene in comparison with the β-carotene of green color *A. gangeticus* [76]. β-carotene of red color *A. gangeticus* was 2 to 3-fold higher than the β-carotene of *A. caudatus* leaves [20]. *A. hypochondriacus* exhibited the maximum TF, TP, and TAC (ORAC and FRAP methods) in comparison with *A. caudatus* [71]. Additionally, they noted the maximum TF, TP, and TAC (FRAP) in comparison with sprouts, seed, flowers, and stalks. The procedures of determination and extraction and standards differed from the present methodology; hence, it’s tedious to compare the current findings with their results. The accessions LS7 and LS9 demonstrated high phenolic profiles, antioxidant constituents such as pro-vitamin A, vitamin C, TP, TF, and AC. The accessions LS7 and LS9 could be used as antioxidant profiles enriched high-yielding varieties. It revealed from the study that these two accessions could offer huge prospects for feeding the antioxidant-deficient community.

Pro-vitamin A and vitamin C had significant positive interrelationships with vitamins, TP, TF, AC (DPPH and ABTS) that signify that both pro-vitamin A and vitamin C exhibited good antioxidant potentiality. The results of the present study corroborated with the results of our earlier study of drought and salt-stressed *A. tricolor* [22–24, 28]. The significant correlations among TP, TF, AC (DPPH and ABTS) were observed that indicated high potential antioxidant activity of TP and TF in *A. gangeticus*. The correlations of TP and TF, versus AC (FRAP) in salt-stressed purslane [77], were corroborative to our present findings. Similarly, AC (ABTS) was significantly associated with AC (DPPH) that validated the estimation of antioxidant activity of two different methods in *A. gangeticus*.

## Conclusions

Twenty-five flavonoids and phenolic acids such as *p*-hydroxybenzoic acid, salicylic acid, protocatechuic acid, vanillic acid, gentisic acid, gallic acid, β-resorcylic acid, ellagic acid, syringic acid, chlorogenic acid, *m*-coumaric acid, *trans*-cinnamic acid, caffeic acid, ferulic acid, sinapic acid, *p*-coumaric acid, naringenin, rutin, isoquercetin, kaempferol, catechin, hyperoside, myricetin, apigenin, and quercetin were identified in the *A. gangeticus* genotypes. *A. gangeticus* genotype LS7 and LS9 had abundant phenolic acids, flavonoids, antioxidant constituents, and antioxidant potentiality. It revealed from the correlation study that all antioxidant compositions of *A. gangeticus* exhibited high antioxidant potentiality. It revealed from the study that two *A. gangeticus* genotypes had excellent sources of antioxidants components capable of quenching ROS. It revealed from this study that data obtained from advance lines of *A. gangeticus* genotypes contribute to the scientists to evaluate pharmacologically active constituents.

## Data Availability

All data generated or analyzed during this study are included in this article and available from the corresponding author on reasonable request.

## Abbreviations

ANOVA: Analysis of variance
ABTS: 2,2′-azino-bis-(3-ethylbenzothiazoline-6-sulphonic acid)
AAS: Atomic absorption spectrophotometry
DPPH: 2,2-diphenyl-1-picrylhydrazyl
DHA: Dehydroascorbate
DMRT: Duncan’s Multiple Range Test
DTT: Dithiothreitol
DW: Dry weight
FW: Fresh weight
FRAP: Ferric reducing antioxidant power
GAE: Gallic acid equivalent
HCl: Hydrochloric acid
HPLC-UV: High-performance liquid chromatography-ultra violet
LC-MS-ESI: Liquid chromatography-mass spectroscopy-electrospray ionization
ORAC: Oxygen radical absorbance capacity
RCBD: Completely randomized block design
RE: Rutin equivalent
ROS: Reactive oxygen species
SD: Standard deviation
TAC: Total antioxidant capacity
TEAC: Trolox equivalent antioxidant capacity
TFC: Total flavonoid content
TPC: Total polyphenol content
TPI: Total phenolic index
